# The Assessment of Muscular Effort, Fatigue, and Physiological Adaptation Using EMG and Wavelet Analysis

**DOI:** 10.1371/journal.pone.0135069

**Published:** 2015-08-11

**Authors:** Ryan B. Graham, Mark P. Wachowiak, Brendon J. Gurd

**Affiliations:** 1 School of Physical and Health Education, Nipissing University, North Bay, ON, Canada; 2 School of Kinesiology and Health Studies, Queen’s University, Kingston, ON, Canada; 3 Department of Computer Science and Mathematics, Nipissing University, North Bay, ON, Canada; The University of Queensland, AUSTRALIA

## Abstract

Peroxisome proliferator-activated receptor gamma coactivator 1 alpha (PGC-1α) is a transcription factor co-activator that helps coordinate mitochondrial biogenesis within skeletal muscle following exercise. While evidence gleaned from submaximal exercise suggests that intracellular pathways associated with the activation of PGC-1α, as well as the expression of PGC-1α itself are activated to a greater extent following higher intensities of exercise, we have recently shown that this effect does not extend to supramaximal exercise, despite corresponding increases in muscle activation amplitude measured with electromyography (EMG). Spectral analyses of EMG data may provide a more in-depth assessment of changes in muscle electrophysiology occurring across different exercise intensities, and therefore the goal of the present study was to apply continuous wavelet transforms (CWTs) to our previous data to comprehensively evaluate: 1) differences in muscle electrophysiological properties at different exercise intensities (i.e. 73%, 100%, and 133% of peak aerobic power), and 2) muscular effort and fatigue across a single interval of exercise at each intensity, in an attempt to shed mechanistic insight into our previous observations that the increase in PGC-1α is dissociated from exercise intensity following supramaximal exercise. In general, the CWTs revealed that localized muscle fatigue was only greater than the 73% condition in the 133% exercise intensity condition, which directly matched the work rate results. Specifically, there were greater drop-offs in frequency, larger changes in burst power, as well as greater changes in burst area under this intensity, which were already observable during the first interval. As a whole, the results from the present study suggest that supramaximal exercise causes extreme localized muscular fatigue, and it is possible that the blunted PGC-1α effects observed in our previous study are the result of fatigue-associated increases in muscle acidosis. This should be explored in future research using further combinations of EMG and muscle biochemistry and histology.

## Introduction

Mitochondrial biogenesis is a hallmark adaptive response of skeletal muscle to exercise training [1], and therefore the study of factors controlling biogenesis is critical to our understanding of this multi-factorial adaptive response. Peroxisome proliferator-activated receptor gamma coactivator 1 alpha (PGC-1α) is a transcription factor co-activator that helps coordinate this response within skeletal muscle [2]. Specifically, PGC-1α is activated following exercise, triggering the induction of gene transcription and eventually protein synthesis for mitochondrial protein [3]. Given the importance of mitochondrial content for both exercise performance and health [4], the study of the physiological factors that control the activation and up-regulation of PGC-1α represents an important area of research.

While evidence gleaned from submaximal exercise (exercise performed at work rates that are less than maximal aerobic power) suggests that both intracellular pathways associated with the activation of PGC-1α, as well as the expression of PGC-1α itself are activated to a greater extent following higher intensities of exercise [5], we have recently shown that this effect does not extend to supramaximal exercise [6]. Specifically, we observed a reduction in the expression of PGC-1α following supramaximal (133% of maximal aerobic power) compared to maximal (100% of maximal aerobic power) exercise, despite elevated surface electromyography (EMG) amplitude-derived estimates of overall muscle activation, recruitment, and force generation [6]. As the mechanisms underlying this apparent blunting of PGC-1α activation following supramaximal exercise remain unclear, further investigation of the factors that may contribute to this response is needed.

Spectral analyses of EMG data may provide a more in-depth assessment of changes in muscle dynamics occurring across different exercise intensities than more traditional, amplitude-derived estimates of muscle activation [7]. During voluntary muscle contractions of increasing intensity, neural drive is altered to increase motor unit firing frequency (rate coding) and/or to increase the number of recruited motor units [8]. Since surface EMG measures the sum of motor unit action potentials (MUAPs) firing at specific frequencies [9,10], spectral analyses can decompose this time-varying signal into its frequency components. While somewhat controversial, results derived from spectral analysis have been associated with a variety of factors including fibre type (i.e. Type I vs. II), conduction velocity, and muscular fatigue [11].

Several different methods exist to assess the spectral distributions of EMG data [8]. The most frequently applied of these methods, the Fourier transform (FT), is not well suited to dynamic muscular contractions where the signal is non-stationary due to alterations in muscle force, contraction velocity, and length [8,10]. While this limitation can be mitigated by identifying short overlapping time windows in which the EMG signal does not change its cycle-stationarity, subsequently extracting the frequency content via Fourier techniques, this process, known as the Short-Term Fourier Transform (STFT) [12], also possesses significant limitations [8,10]. The STFT allows only fixed time-frequency resolution, with good frequency resolution possible with longer time windows. However, the non-stationary characteristics of EMG bursts are typically of very short duration; usually less than 500 milliseconds [12].

Consequently, alternative time-frequency methods, most notably wavelet approaches, have been gaining popularity for assessing the spectral content of surface EMG during dynamic contractions [12–16]. Wavelet transforms (WTs) decompose the signal in both frequency and time onto a series of basis functions whose characteristics can be specified according to analysis needs [8,10]. The most suitable type of WT for surface EMG is the continuous wavelet transform (CWT), as CWTs allow for freely selectable wavelet scales corresponding to frequency and time values, thereby providing fine control over frequency resolution [7–10,17].

Because of these advantages, the current study applied quantitative CWT analysis to our previously collected surface EMG data recorded from the vastus lateralis muscle [6], in order answer several primary research questions. First, we were interested in assessing whether wavelet analyses of EMG data can detect differences in muscle electrophysiological properties (e.g. frequency characteristics related to fibre type (i.e. Type 1 vs. II) and conduction velocity [11]) at different exercise intensities (i.e. 73%, 100%, and 133% of peak aerobic power (VO_2_peak)). Second, we wanted to assess changes in muscular effort and fatigue across a single interval of exercise at each intensity. The overall goal of this study was to shed mechanistic insight into our previous observation that the increase in PGC-1α is dissociated from exercise intensity following supramaximal exercise [6]. To the best of our knowledge, this is the first study to directly compare EMG-based spectral approaches with associated molecular and biological measures, and the first study to assess changes in wavelet-based spectral EMG measures during supramaximal exercise.

## Materials and Methods

### Experimental Approach

To assess the changes in the spectral properties of surface EMG associated with differential exercise intensity and changes in mRNA expression of PGC-1α, participants performed high-intensity interval exercise (HIIE) targeting 73, 100, and 133% of their peak aerobic power on 3 separate occasions. All interval sessions were matched for total work (kJ). Muscle activation was continuously monitored during each interval of each session using surface EMG, and the underlying frequency characteristics were extracted at each point in time using continuous wavelet transforms. The present work is part of a larger study, and data regarding participant and exercise characteristics, along with changes in EMG activation amplitudes and gene expression of PGC-1α and its regulators, have already been published [6].

### Participants

Lean healthy men (*n* = 8; 22 ± 2 yrs; 23 ± 2 kg/m^2^; 53 ± 6 ml/kg/min) volunteered to participate in the present study. Due to problems with the EMG signals from one participant, the data from only 7 participants were subjected to all analyses outlined below. All participants were recreationally active but were not involved in a specific training program at the time of recruitment. The experimental protocol and associated risks were explained both orally and in writing to all participants before written consent was obtained. The study was approved by the Health Sciences Research Ethics Board at Queen’s University.

### VO_2_peak Test and HIIE Protocols

A detailed description of the VO_2_peak test and exercise protocols are provided in Edgett et al. (2013) [6]. During their initial visit to the laboratory, participants’ anthropometrics were measured and they performed a VO_2_peak test on a cycle ergometer (Monark, Ergomedic 874E, Varberg, Sweden) with gas exchange being collected continuously throughout the test (Moxus, AEI Technologies, Pittsburgh, PA). The test protocol consisted of loadless pedaling for five minutes at 80 RPM, a step increase to 80 watts for one minute and subsequent increases in work rate of 25 watts per minute until volitional fatigue. During the VO_2_peak test participants were familiarized with a restraint system intended to standardize leg position, maintain posture, and to prevent participants from standing during the subsequent HIIE visits.

For all experimental visits, participants were instructed to refrain from exercise for 24 hours prior to their visit, and arrived to the lab at 8:00 am after having fasted for 12 hours. Following a standardized breakfast (toasted bagel [~190 kcal; 1 g fat, 36 g carbohydrate, 7 g protein] with 15 g of cream cheese [~45 kcal; 4 g fat, 1 g carbohydrate, 1 g protein]), participants rested in a seated position for one hour after which the first of two muscle biopsies were obtained from the vastus lateralis muscle as close as possible to the recommended EMG placement locations from SENIAM (Surface EMG for Non-Invasive Assessment of Muscles). Following this first biopsy, participants were moved to the cycle ergometer where EMG electrodes were applied over the vastus lateralis of the leg opposite to the one that had been biopsied at the predetermined location. Participants then performed HIIE at a target work rate (WR) of 73, 100, or 133% of their peak aerobic power (highest 30 second power output [watts] from the VO_2_peak test). Following completion of HIIE, participants rested in a seated position for three hours before a second muscle biopsy sample was taken from close proximity (approximately 10–20 mm) to the first biopsy on the same leg.

Following a randomized crossover design, each participant performed three different HIIE protocols on a cycle ergometer (Monark, Ergomedic 874E, Varberg, Sweden) separated by a washout period of approximately one week (minimum of 6 days and maximum of 2 weeks). All interval protocols consisted of a five minute loadless warm up, followed by HIIE consisting of one minute intervals separated by one minute of loadless cycling at a cadence of the participants choosing on a cycle ergometer. During the intervals, a load was added to the bike such that cycling at 80 rpm would result in a WR of 73, 100, or 133% of peak aerobic power. These intensities were chosen so that a matched amount of external work would be achieved in 11, 8 and 6 intervals for each intensity, respectively. Subjects were instructed to maintain 80 rpm at all times; however, when rpm fell below 80, additional intervals were added such that the target amount of work (± ~5%) was achieved in all HIIE sessions (See Edgett et al., 2013 Table 2 for details). Throughout all intervals, RPM and work rate were continuously monitored using a magnetic switch with the components secured to the ergometer crankshaft and body. Starting leg for biopsies/EMG was randomized for each participant, and leg was altered between HIIE protocol days. Only EMG data from the first interval were analyzed here.

### EMG Procedures

Vastus lateralis muscle activity was monitored unilaterally using pre-gelled, self-adhesive, bipolar Ag/AgCl EMG electrodes (EasyTrode 3SG3-N, MultiBioSensors, El Paso, TX, USA). The electrodes were placed longitudinally with respect to the underlying muscle fibre arrangement, as per the recommendations of SENIAM [18]. Before the electrodes were applied, the skin was shaved and abraded with alcohol to minimize impedance, and wires were subsequently well-secured to avoid movement-induced artefacts [19]. Raw EMG signals were bandpass filtered (10–1000 Hz) and amplified (AMT-8, Bortec Biomedical Ltd., Calgary, AB, Canada; input impedance = 10 GΩ, CMRR = 115 dB at 60 Hz), and then captured digitally concurrent to the switch data at 2048 Hz using a USB-6008 A2D board and custom Labview software (National Instruments, Austin, TX, USA). To account for differences in electrode placement and EMG characteristics (e.g. skin impedance) between days, a series of three seated maximum voluntary contractions (MVC’s) were performed prior to testing for EMG normalization.

### Signal Processing

**Fig 1:**
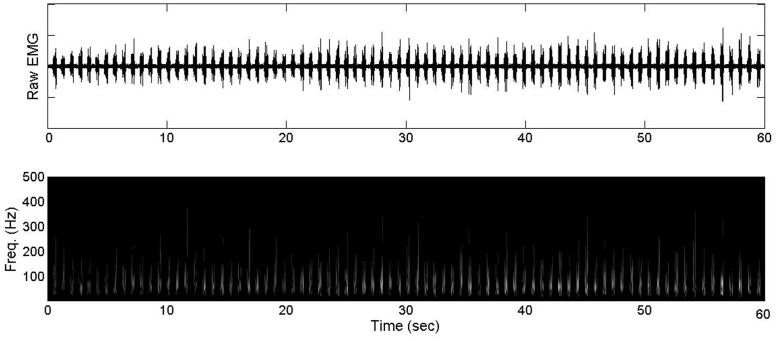
Exemplar raw EMG data as well as Morlet continuous wavelet transforms across one entire 60 second interval.

As noted earlier, the time-frequency characteristics of wavelet analysis were exploited so that the EMG frequencies could be analyzed as a function of percent cycle (a time-based measurement), an approach that is not possible with the Fourier transform. This was done after normalizing the EMG data from each day to the average root mean squared value across the three MVC’s. A variety of wavelet methods, both continuous [7,13,16,20] and discrete [12], can be employed for this task. Continuous wavelet methods were employed in the current work to analyze signal characteristics over a broad dynamic range. Furthermore, the CWT can be considered as a 2D “map” from which morphological features (e.g. easily distinguished areas of high power) can be determined through standard image processing techniques (Fig 1). Several choices for wavelet basis functions are available for analyzing the specific characteristics of surface EMG. For example, some investigators have used a filter bank of Cauchy wavelets to separate simultaneous events in time and frequency in surface EMG [20]. Others have used windowed sinusoids to select specific frequencies [21]. However, the current study employed complex Morlet wavelets *ψ*(*t*), where the mother wavelet, as a function of time *t*, is given as:

$$\psi (t)=\frac{1}{\sqrt{b\pi }}e^{{-\left(\frac{t}{b}\right)}^{2}}e^{j2\pi f_{c}t},$$ (1)

where $j=\sqrt{-1}$, *f*_c_ is the centre frequency of *ψ*(*t*) (the position of the global maximum of the Fourier transform Ψ of the mother wavelet *ψ*), and *b* > 0 is the bandwidth parameter. In the present study, the bandwidth parameter and centre frequency were set to 1. The Morlet wavelet was used because of its straightforward relationship between wavelet scale (*s*) and frequency (*f*) [17] given the sampling frequency (*f*_s_): *f* = *f*_s_ / *s*, and because of its accepted use in EMG analysis [14,22–24]. The Morlet filter bank is also mathematically related to the widely-used Cauchy wavelets [25].

Frequencies ranged from 5 to 500 Hz, in increments of 1 Hz. The CWT power was computed as the squared magnitude. For each wavelet power spectrum, the first and last ten (10) cycles were extracted within the first interval under each intensity condition. Those parts of the CWT were then re-mapped with 2D cubic interpolation to percentage of cycle with 401 intervals (units of 0.25%). In subsequent analysis, the CWT bursts were averaged for the first and last ten cycles. This resulted in a CWT with one major frequency component at the burst. Wavelet transforms and analyses were performed in the MATLAB programming environment (The MathWorks, Natick, MA, USA).

To gather further statistics, the components of each CWT were classified into four categories with Otsu’s segmentation method, which is a histogram-based image segmentation algorithm that determines thresholds that minimize intra-category variance by iteratively computing category probabilities and means [26]. To reduce artifacts, the fourth class (with the highest powers) was selected for subsequent analysis, which included: (1) the major frequency component of each burst, computed as the weighted centroid (centroid of the burst region weighted by the distribution of powers within the region); (2) the mean power for the highest power region, and; (3) the area of the burst (measured in pixels of the CWT). The burst area on the 2D CWT indicates the range of frequencies (vertically on the map) in which high power was detected, and the duration, providing a quantitative way to distinguish between conditions and intervals. Larger areas may indicate a wider frequency band, which could correlate to a larger recruitment of motor units and muscle fibres. A wider area along the time axis may suggest recruitment of muscle fibres during the push and pull phases of cycling. In cases where the segmentation resulted in multiple disconnected bursts in the highest-power class, the burst for analysis was chosen on the basis of either maximum area or highest mean power within the burst. However, both of these approaches gave the same results, suggesting that either the maximum area or highest mean power is a suitable metric for selecting bursts. Consequently, only the maximum area results are reported here.

Additionally, for visual comparison, the 2D CWT powers were normalized to the 133% (maximum intensity) condition, and plotted for the first 10 and last 10 cycles within the first interval for each intensity condition. The normalization was achieved by dividing the 73%, 100%, and 133% intensities by the total power of the 133% transform so that the total power of the 133% intensity for each interval is unity. To further analyze frequency changes across conditions, the 2D transform was integrated over time. This approach is different than the weighted centroid measurement described above, as the latter takes entire area and subtle frequency components in burst into account, while a power-vs.-frequency analysis shows the most powerful components strictly on the basis of power. From the resulting power vs. frequency function, the median power frequency was calculated with standard methods.

### Statistical Analysis

For each dependent variable (work rate, main burst frequency, mean burst power, burst area, and median power frequency) the effects of exercise intensity were analyzed in several ways. To address the first goal of detecting differences in muscle electrophysiological properties at the different exercise intensities, data from the first 10 cycles within the first interval (fresh muscle state) for each intensity were compared using Friedman’s non-parametric rank test with Dunn’s post-hoc multiple comparisons (α = 0.05) (GraphPad Prism V 5.01, GraphPad Software, Inc., La Jolla, CA, USA). To address the second goal of determining changes in muscular effort and fatigue at each intensity, change scores were computed for each dependent variable across the first interval (data from last 10 cycles—data from first 10 cycles); these change scores were also analyzed using Friedman’s rank test with Dunn’s LSD post-hoc comparisons (α = 0.05). This non-parametric equivalent of repeated-measures ANOVA was used due to the small sample size (n = 7).

## Results

**Fig 2:**
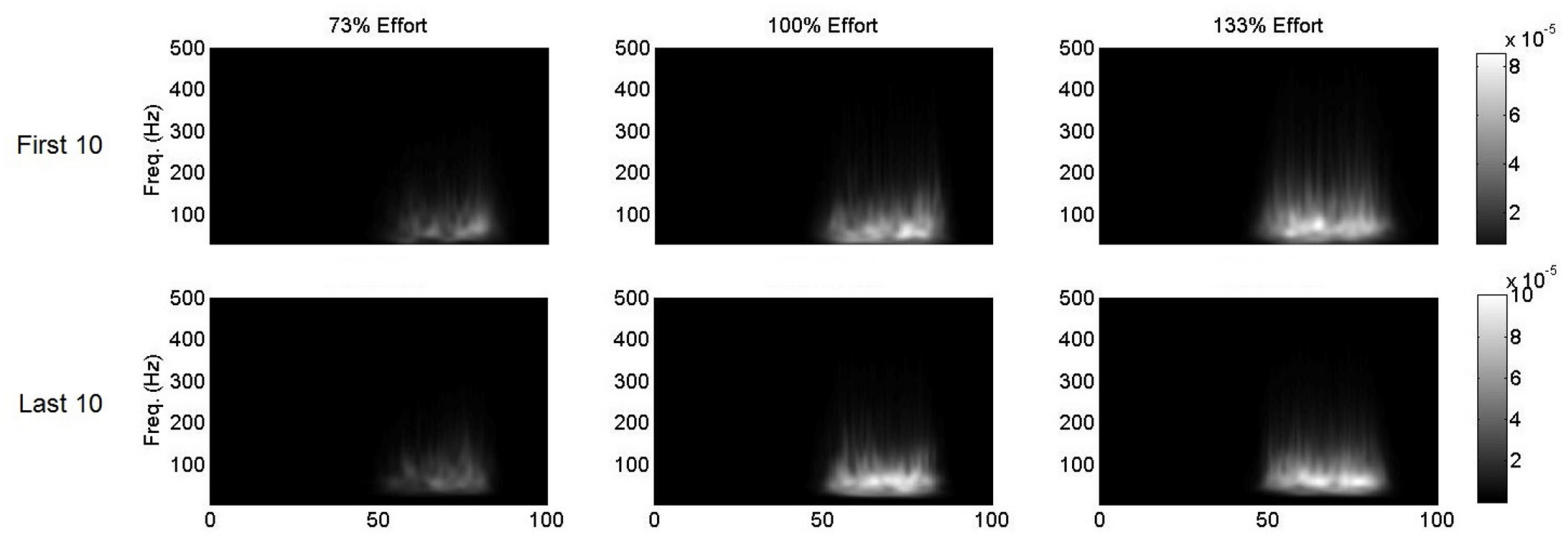
Morlet continuous wavelet transforms averaged across the first 10 and last 10 cycles in the first interval at each intensity level. In all cases the time scale is normalized from 0 to 100% of the pedal cycle, and the power scale is normalized to the 133% intensity condition.

**Fig 3:**
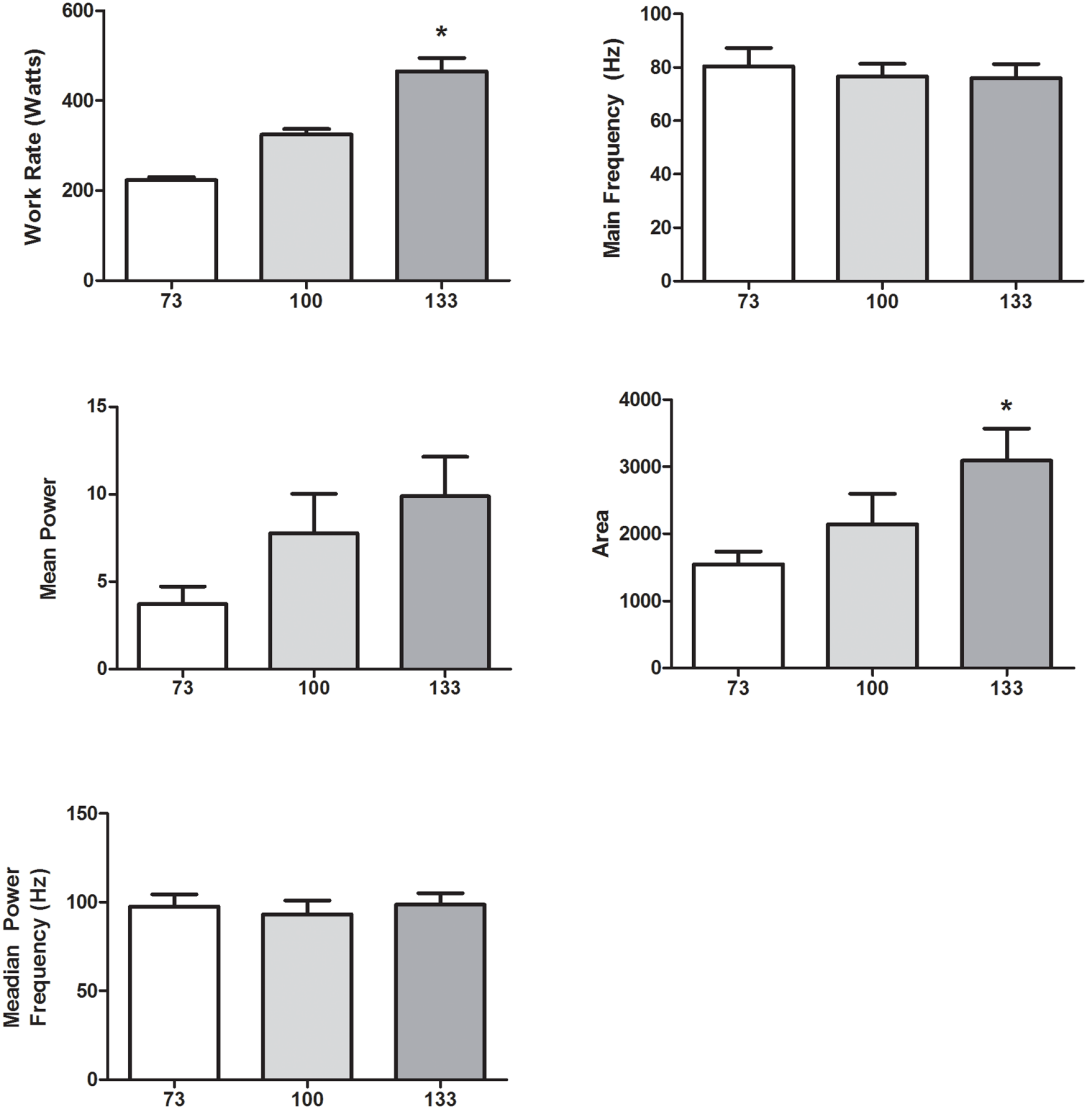
Work rate (RPM * load on the bike), main burst frequency, mean power, burst area, and median power frequency results for the first 10 cycles in the first interval at each intensity level. * Sig from 73%.

**Fig 4:**
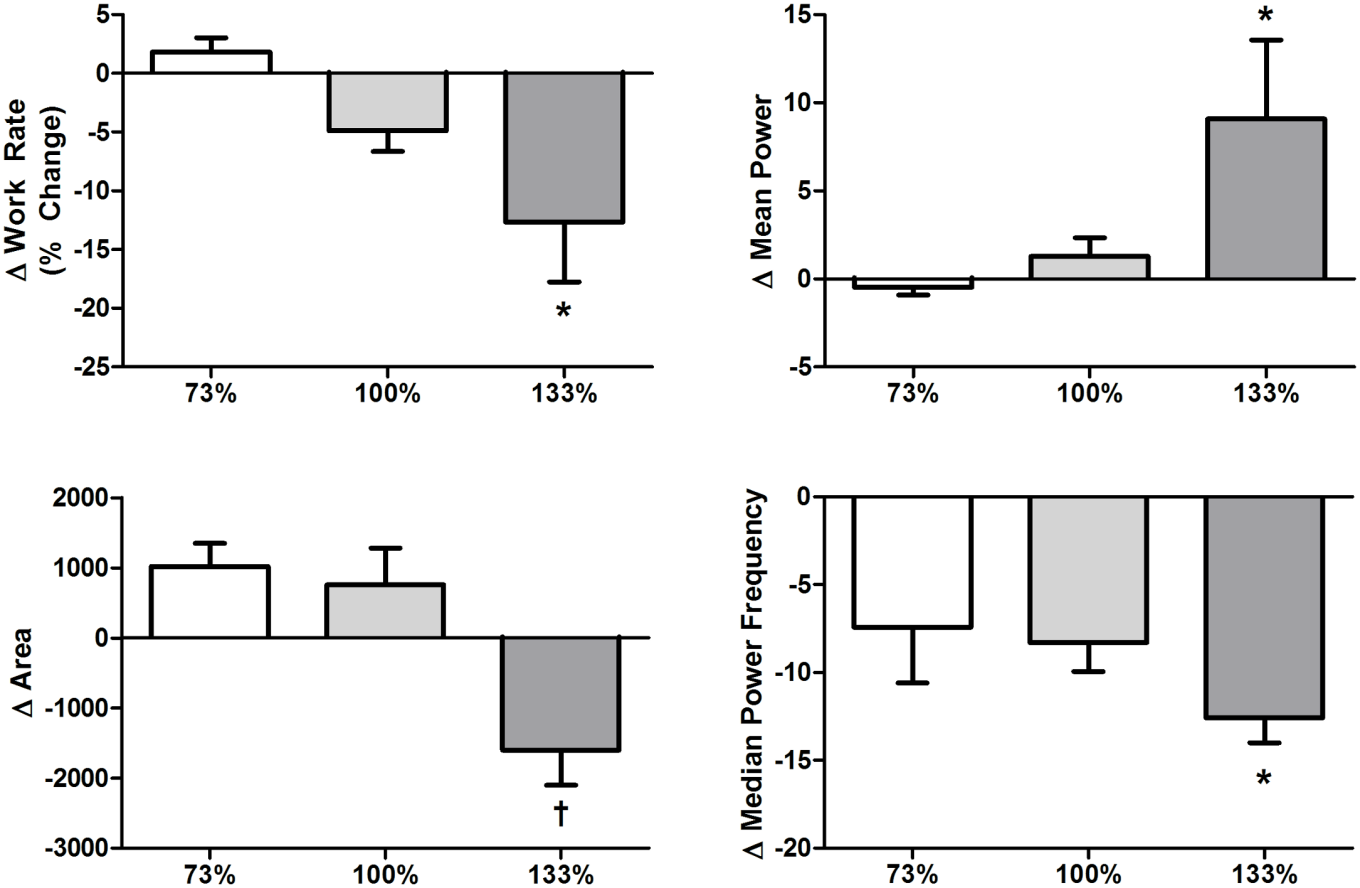
Change in work rate, burst mean power, burst area, and median power frequency across the first interval at each intensity level. Note: * Sig from 73%, † sign from 73% and 100%.

Averaged Morlet wavelet transforms normalized to the 133% condition for all intensities (interval 1) are displayed in Fig 2. All quantitative analyses are summarized in Figs 3–4.

In terms of the first goal of the study, there was a significant main effect of exercise intensity on increasing work rate (p = 0.001) and burst area (p = 0.012) during the first 10 cycles of the first interval (Fig 3). In both cases, post-hoc testing revealed that the 133% condition was significantly greater than the 73% condition (p<0.001 and p = 0.003, respectively), but not the 100% condition (p = 0.061 in both cases). The 73% condition was also not significantly different from the 100% condition (p = 0.061 and 0.285, respectively). There was no significant main effect of intensity on main burst frequency (p = 0.867), mean power (p = 0.867), or median power frequency (p = 0.540).

In terms of the second goal of the study, there was a significant main effect of exercise intensity on changes in work rate (p = 0.050), mean power (p = 0.028), burst area (p = 0.018), and median power frequency (p = 0.034) across the first the interval (Fig 4). For work rate, there were larger drop-offs in work rate with higher exercise intensities (73% vs. 100% p = 0.423, 73% vs. 133% p = 0.016, 100% vs 133% p = 0.109). For mean power there were larger increases in power with higher intensities (73% vs. 100% p = 0.181, 73% vs. 133% p = 0.008, 100% vs 133% p = 0.181). For median power frequency there were larger decreases in frequency with higher intensities (73% vs. 100% p = 0.285, 73% vs. 133% p = 0.011, 100% vs 133% p = 0.142). Lastly, there were similar increases in area for the 73% and 100% exercise intensities, but a large drop off in area for the 133% exercise intensity (73% vs. 100% p = 0.593, 73% vs. 133% p = 0.003, 100% vs 133% p = 0.033).

## Discussion

The goals of the present study were twofold. First, we were interested in assessing whether wavelet analyses of EMG data can detect differences in muscle electrophysiological properties (e.g. frequency characteristics related to fibre type (i.e. Type 1 vs. II) and conduction velocity [11]) at different exercise intensities (i.e. 73%, 100%, and 133% of peak aerobic power (VO_2_peak)). Second, we wanted to assess changes in muscular effort and fatigue across a single interval of exercise at each intensity. The overall goal of this study was to shed mechanistic insight into our previous observations that the increase in PGC-1α is dissociated from exercise intensity following supramaximal exercise. [6].

In general, the CWTs revealed that supramaximal exercise (133%) did not change muscle fibre recruitment but was the only condition to result in significantly greater levels of localized fatigue than the 73% condition, which directly matched the work rate results. Specifically, there were greater drop-offs in frequency, larger changes in burst power, as well as greater changes in burst area under this intensity, which were already observable during the first interval. As a whole, the results from the present study suggest that these CWT analyses are capable of discerning differences in muscle fatigue across a range of exercise intensities, and that supramaximal exercise causes extreme localized muscular fatigue. These findings and their implications are discussed in greater detail below.

### Continuous Wavelet Transform Differences between Intensities in Unfatigued Muscle States

Based on the visual comparison of CWTs across intensity conditions as well as the quantitative analysis of the burst area results, there were corresponding increases in burst area with increasing intensity conditions during the first 10 cycles of the first interval. This increase in burst area is expected, since the actual work rate was higher, thereby requiring more muscle activity, agreeing with the amplitude-based EMG findings from our previous work [6], where there was a near-linear increase in muscle activation with intensity level. In terms of signal frequency content, there was no significant main effect of intensity condition and either frequency measure (main frequency or median power frequency), and therefore we cannot confirm whether there was differential fibre type recruitment between the varying intensity conditions (i.e. higher type 2 fibre type recruitment with higher intensity). Huber et al. (2010) reported lower frequencies in strength trained athletes who should, in theory, have a higher percentage of type 2 fibres. Previous findings have also shown greater motor unit synchronization at higher intensities, which leads to an increase of power in the EMG spectra and a downshift in frequency content [7,27]. The lack of differences may be explained by the relatively high percentage of type I fibres in the vastus lateralis [28], as it has been previously reported that muscles with a greater percentage of type I fibres may not display shifts in the power spectrum based on increased force levels, despite additional recruitment of MU during maximal contractions [29]. The relationship between fibre type recruitment and spectral properties should be directly investigated in the future using molecular and EMG approaches.

### Continuous Wavelet Transform Differences between Intensities with Localized Fatigue

Based on visual inspection of the CWTs as well as the quantitative analyses, there were significantly larger reductions in burst area across the first interval under the 133% condition when compared to either other condition. Moreover, there were larger increases in mean power with increasing exercise intensity (the 133% condition was the only condition significantly different than the 73% submaximal condition). These results, when combined with the drop-offs in work rate observed at the higher intensity (again the 133% condition was the only condition significantly different than 73%) suggest that supramaximal exercise induces greater amounts of localized muscle fatigue than either other condition. This is further confirmed by the significant main effect of exercise intensity on changes in median power frequency, as it is well known that downward shifts in the frequency domain occur with localized muscular fatigue (e.g. [10]).

### The Relationship Between CWT and Molecular Adaptations

In our previous work, we observed a dissociation between the post-exercise increases in PGC-1α and both exercise intensity and EMG derived estimates of muscle activation [6]. While in our original publication we were unable to comment with certainty what the mechanisms underlying this dissociation were, the current analysis suggests that the blunted increase in PGC-1α observed following intervals at 133% may have resulted from increased localized muscular fatigue rather than from differential fibre type recruitment. In support of this contention, there is some evidence that decreases in intramuscular pH may blunt the adaptive response to exercise in skeletal muscle.

pH is known to decrease to a greater extent during higher intensities of exercise [30], with lower pH associated with muscle fatigue in muscle [31]. Evidence from both rats [32] and humans [33] suggests that reducing the decrease in pH associated with high intensity exercise can improve the adaptive responses to exercise. Taken together with the data from the current study, these earlier works imply that both the elevated fatigue and blunted PGC-1α response [6] observed following supramaximal exercise may have been the result of increased intramuscular acidosis. Thus, the information provided by CWT analysis appears to have provided valuable mechanistic insight into our previous observations, supporting the integrative use of both molecular approaches and EMG in future studies.

### CWT Limitations

Although areas and shape measurements of the EMG bursts in the wavelet transform provide quantifications of features present in the CWT, they must be used cautiously. These metrics are susceptible to artifact influence (e.g. spectral leakage caused by the discrete implementation of the continuous wavelet windowing function). Such influence, however, is reduced by using the highest-power class after segmenting the power spectrum into four classes. This high-power class represents the main area of burst activity in the spectrum, as observed empirically.

Interpreting the maximum wavelet power—and the frequency at which the maximum power is found—on the basis of EMG bursts in the CWT is also affected by the same artifacts just mentioned. Consistent with determining the main power and frequency components of CWT bursts by maximal values of regions of interest [34,35] identifying these features with the weighted centroid, or centre of gravity, of the segmented bursts is justified, as using the maximum power region is more robust than simply considering the characteristics of numerically maximal powers.

EMG interpretation in the wavelet domain is heavily influenced by the choice of wavelet function [36]. As an alternative to the Morlet CWT, intensity analysis using Cauchy wavelets [20]–which provide an optimal filter bank that is discrete in frequency but continuous in time—is increasingly used for EMG processing [37,38]. Consequently, comparative analysis will be conducted using these wavelets in future work. Additionally, alternative measures not directly related to features in 2D CWT map may be used. For instance, quantifying spectral features through rigorous pattern recognition approaches (e.g. with principal component analysis [39]) is a promising direction.

## Conclusion

In conclusion, the results presented in this paper indicate that supramaximal exercise exhibits the greatest amount of localized muscular fatigue, which may suggest that our previously observed reduction in the expression of PGC-1α following supramaximal compared to maximal exercise, despite elevated surface electromyography (EMG) amplitude-derived estimates of overall muscle activation, may simply be due to exceptional amounts of fatigue. Therefore, we speculate that the reduced increase in PGC-1a following supramaximal exercise observed previously [6] may have resulted from increases in intramuscular acidosis associated with high-intensities of exercise and muscle fatigue. Future work will further investigate this question using a combination of molecular and EMG techniques, and will also attempt to shed further insight into whether fibre type recruitment is differentially affected during supramaximal exercise. Work that incorporates neuromuscular tests such as percutaneous neuromuscular electrical stimulation (e.g. twitch amplitude, half-relaxation, M-Wave peak-to-peak and duration, and so on) to detect, better distinguish, and quantify potential central (nervous component) or peripheral (structural) fatigue would also be beneficial.
